# Particle Bombardment of the *cry2A* Gene Cassette Induces Stem Borer Resistance in Sugarcane

**DOI:** 10.3390/ijms19061692

**Published:** 2018-06-06

**Authors:** Shiwu Gao, Yingying Yang, Liping Xu, Jinlong Guo, Yachun Su, Qibin Wu, Chunfeng Wang, Youxiong Que

**Affiliations:** Key Laboratory of Sugarcane Biology and Genetic Breeding, Ministry of Agriculture and Key Laboratory of Crop Genetics and Breeding and Comprehensive Utilization, College of Crop Science, Fujian Agriculture and Forestry University, Ministry of Education, Fuzhou 350002, China; gaoshiwu2008@126.com (S.G.); yingyingyang13@163.com (Y.Y.); jl.guo@163.com (J.G.); syc2009mail@163.com (Y.S.); wqbaidqq@163.com (Q.W.); 18305999305@163.com (C.W.)

**Keywords:** sugarcane, *cry2A* gene, particle bombardment, stem borer, resistance

## Abstract

Sugarcane borer is the most common and harmful pest in Chinese sugarcane fields, and can cause damage to the whole plant during the entire growing season. To improve borer resistance in sugarcane, we constructed a plant expression vector pGcry2A0229 with the *bar* gene as the marker and the *cry2A* gene as the target, and introduced it into embryogenic calli of most widely cultivated sugarcane cultivar ROC22 by particle bombardment. After screening with phosphinothricin in vitro and Basta spray, 21 resistance-regenerated plants were obtained, and 10 positive transgenic lines harboring the *cry2A* gene were further confirmed by conventional PCR detection. Real-time quantitative PCR (RT-qPCR) analysis showed that the copy number of the *cry2A* gene varied among different transgenic lines but did not exceed four copies. Quantitative ELISA analysis showed that there was no linear relationship with copy number but negatively correlated with the percentage of borer-infested plants. The analysis of industrial and agronomic traits showed that the theoretical sugar yields of transgenic lines TR-4 and TR-10 were slightly lower than that of the control in both plant cane and ratoon cane; nevertheless, TR-4 and TR-10 lines exhibited markedly lower in frequency of borer-infested plants in plant cane and in the ratoon cane compared to the control. Our results indicate that the introduction of the *cry2A* gene via bombardment produces transgenic lines with obviously increased stem borer resistance and comparable sugar yield, providing a practical value in direct commercial cultivation and crossbreeding for ROC22 has been used as the most popular elite genitor in various breeding programs in China.

## 1. Introduction

Sugarcane is the most important sugar crop, with sucrose accounting for 80% of the total sugar production in the world and accounting for more than 92% of total sugar production in China. As a C_4_ crop, sugarcane makes it one of the most important energy crops due to its high biomass, high fiber, and years of ratooning. Nearly 90% of biofuel ethanol is produced by sugarcane in the United States and Brazil [1]. The risk level of transgenic safety for sugarcane is low mainly due to the following three reasons. Firstly, sugarcane is an asexually propagated crop and generally does not bloom during field cultivation in China, indicating little chance of exogenous gene drift by flowering. Secondly, as an industrial raw material for sucrose and fuel ethanol, sugarcane is not a directly circulated food. The processing of sucrose requires as high as 107 °C for crystallization and crystal sucrose belongs to a purified carbohydrate, without protein ingredient. Besides, the fuel ethanol is not edible. A similar opinion of high food safety level of transgenic sugarcane can be ascribed to the decomposition of protein expressed by the exogenous gene during process of sucrose crystallization [2]. Thirdly, transgenic sugarcane does not affect the microbial community diversity and has no significant effect on enzyme activities in rhizosphere soil, which means better ecological security [3]. To date, two cases involving genetically modified sugarcanes were approved for commercial planting, namely drought resistant transgenic sugarcane in Indonesia and insect-resistant transgenic sugarcane in Brazil.

There are currently five major species of stem borer thriving in Chinese sugarcane fields: *Chilo sacchariphagus* Bjojer, *Scirpophaga nivella* Fabricius, *C. infuscatellus* Snellen, *Argyroploce schistaceana* Snellen, and *Sesamia inferens* Walker. Because several generations occur in one planting season, together with several different species and overlap among generations, stem borer is the most common and harmful pests in the Chinese sugarcane industry. The percentage of dead heart seedlings is normally within the range of 10–20%, but can reach 60% in severely infected sugarcane fields, and the damage incurred during the mid-late stage leads to a significant reduction in sucrose content and the increasing of wind broken stalks [4,5]. Sugarcane, which is heterogeneous polyploid and aneuploid, has a complex genetic background [6,7]. As many as 120 chromosomes are available in modern sugarcane hybrids and there is a lack of stem borer resistance genes in the gene pool [8,9], it is extremely difficult to breed a cultivar with resistance to stem borers in traditional crossbreeding program. Chemical pesticides have long been the main method for preventing and controlling stem borers in Chinese sugarcane, which not only increases production cost but also pollutes the environment.

In 1987, Vaeck et al. successfully introduced the *cry1A(b)* gene into tobacco through *Agrobacterium*-mediated transformation and obtained stem borer-resistant transgenic tobacco [10]. Subsequently, the *Bt* gene was introduced into crops such as cotton [11,12,13,14], maize [15], rice [16,17,18,19], and tomato [20], resulting in effective improvement in stem borer resistance. In sugarcane, Arencibia et al. first described the transformation of *cry1A* gene to improve stem borer resistance in 1997 [8], followed by numerous reports on the improvement of insect resistance in transgenic sugarcanes such as *cry1A(b)* [21,22,23], *GNA* [24,25], *cry1Aa3* [26], *cry1Ac* [27,28,29] and proteinase inhibitor [30,31]. Besides, researchers also attempted to use RNAi technology to control pest damage in sugarcane [32], with success in other crops [33,34,35]. Our previous study showed that the application of insect-resistant transgenic sugarcane can economically and effectively solve the problem of stem borers in the sugarcane industry [36]. *cry2A*, which has low homology (<45%) with *cry1A*, is another Bt protein. Previous researchers demonstrated that cry2A protein is toxic to several lepidopteran pests, indicating its feasibility to be used as a bio-insecticide [37,38]. However, there is no report about the application of *cry2A* in sugarcane.

Compared to other screening marker genes such as *npt II*, *bar* gene screening can be performed at the early stage of genetic transformation of sugarcane, thereby reducing the workload involving tissue culture and increasing efficiency [39]. Thus, the *bar* gene as a screening marker gene has an obvious advantage in eliminating of pseudotyped transformants during selection of putative transformants after bombardment, as sugarcane is phosphinothricin (PPT)-sensitive. The antibiotics and PPT resistance screening tests of two sugarcane genotypes, FN81-745 (*Saccharum* spp. hybrid) and Badila (*Saccharum officinarum*), showed that the effective concentration of both G418 and Hyg was 30.0 mg/L, while only 0.75 mg/L and 1.0 mg/L for PPT, respectively [39]. In plant genetic transformation, the *bar* gene has been widely used as a screening marker gene [40,41], and its application to sugarcane has also been described in several reports [22,29,42,43].

In the present study, to obtain *cry2A* transgenic sugarcane, a plant expression vector pGcry2A0229 with the *bar* gene as a screening marker and *cry2A* as a target gene was constructed and genetically transformed into sugarcane by particle bombardment. PPT and Basta resistance screening and PCR validation were conducted to confirm the positive *cry2A* gene transgenic sugarcane plants. Then, the copy number of the *cry2A* gene and its protein expression in transgenic lines were determined by Real-time quantitative PCR (RT-qPCR) and quantitative ELISA detection of protein, respectively. Finally, several transgenic lines with better comprehensive traits based on a field survey of industrial and agronomic traits were identified, which provides a transgenic line of potential commercial cultivation and the foundation for crossbreeding of stem borer-resistant traits in sugarcane.

## 2. Results

### 2.1. Construction and Verification of the Plant Vector pGcry2A0229

**Figure 1:**
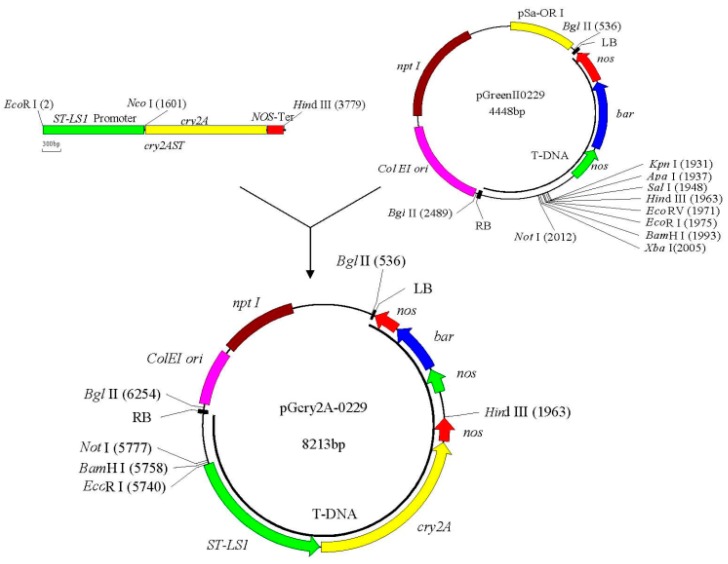
Construction roadmap of the plant expression vector pGcry2A0229.

**Figure 2:**
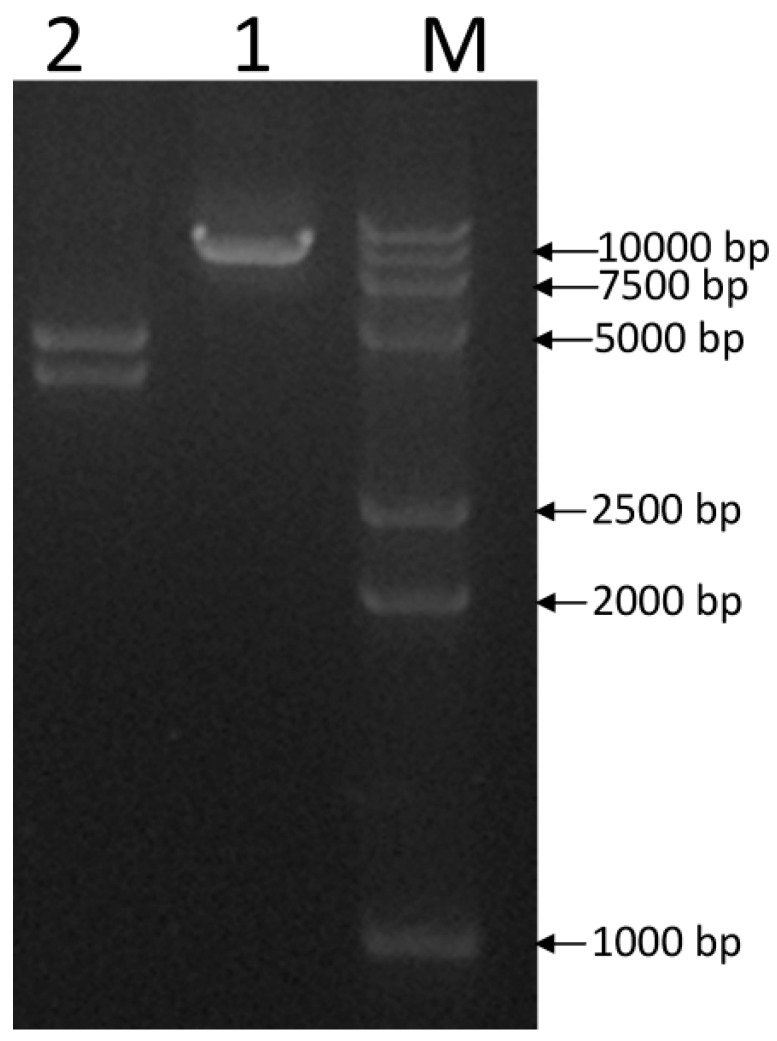
The products of recombinant plasmid pGcry2A0229 digested by restriction enzymes: M, DL15,000 + 2, 000 DNA ladder; 1, The products of pGcry2A0229 digested by *Hin*d III; 2, The products of pGcry2A0229 digested by *Hin*d III and *Eco*R I.

The construction scheme of plant expression vector pGcry2A0229 is depicted in Figure 1. Single-enzyme digestion of pGcry2A0229 using restriction endonuclease *Hin*d III generated the expected single band with a size of 8213 bp. Electrophoresis analysis of double enzyme digestion with *Hin*d III and *Eco*R I showed the expected two bands with sizes of about 4436 and 3777 bp, respectively (Figure 2). The sequencing results confirmed that the pGcry2A0229 was the expected positive recombinant plasmid.

### 2.2. Particle Bombardment and Resistance Screening

**Figure 3:**
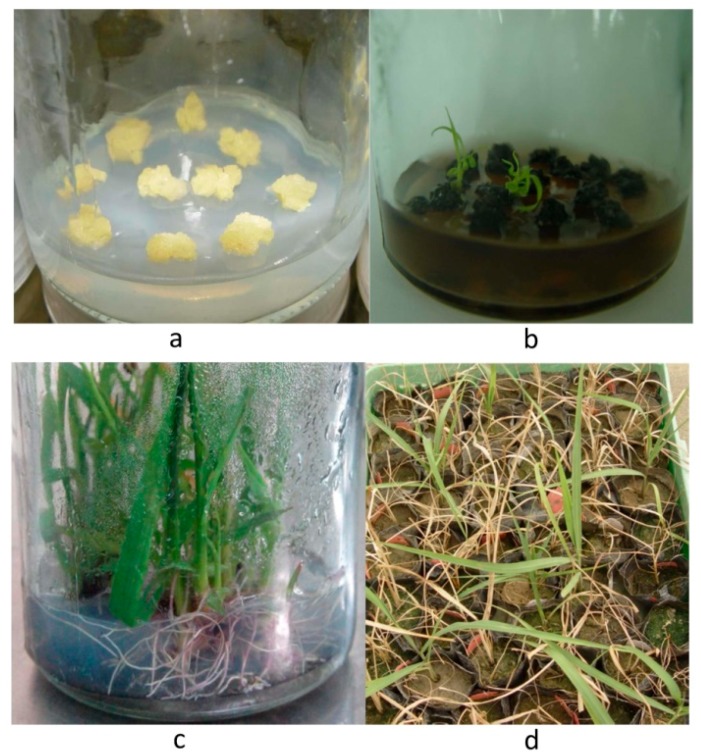
Putative recombinant screening: (**a**) wild-type calli on medium without PPT; (**b**) PPT-resistant plantlets at the differentiation stage on selection medium; (**c**) regenerated plantlets at the stage of rooting culture; and (**d**) spraying screening of resistant plantlets with 3.0‰ Basta.

Micro-bombs were prepared using the tungsten particles and pGcry2A0229 DNA, and bombardment transformation was conducted with the embryogenic calli of sugarcane cultivar ROC22 as the receptor material (Figure 3a). Based on our preliminary experimental results, the embryogenic calli after bombardment was subcultured and differentiated with PPT of 0.8 mg/L. Some tissues gradually differentiated into regenerated plantlets with PPT resistance, whereas most wild-type calli gradually became brown and died (Figure 3b). The negative control died. When the resistant regenerated plantlets grew up to a height of 4–5 cm, they were transferred into the rooting medium without PPT for rooting (Figure 3c). Finally, 95 resistant regenerated plants were obtained.

The resistant regenerated plantlets were transplanted into nutrient pots to ensure their survival. After spray screening with 3.0‰ (*v*/*v*) Basta, most plants gradually turned yellow, wilted, and died after 15–20 days, and finally only 21 plants survived (Figure 3d).

### 2.3. PCR Identification of the cry2A Gene in Resistant Regenerated Plants

**Figure 4:**
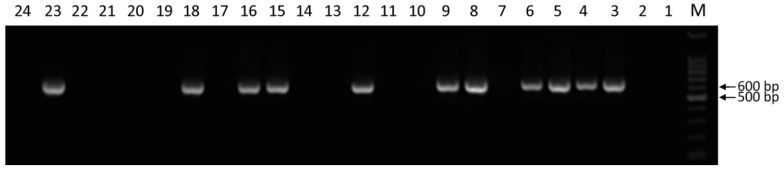
Electrophoretic analysis of PCR amplification products of a putative *cry2A* gene for transgenic sugarcane plants: M, DNA Marker; 1, Blank control of ddH_2_O; 2, Negative control (non-transgenic sugarcane without bombardment); 3, Positive control (plasmid pGcry2A0229); 4–24, Herbicide Basta-resistant plants.

A total of 21 resistant regenerated plantlets obtained by PPT and Basta screening were verified by PCR amplification of the *cry2A* gene. The results showed that a single band was amplified from 10 samples; the position of the band was consistent with that of the positive plasmid and showed an approximate size of 600 bp. The sequencing results were also consistent with the partial sequence of the *cry2A* gene, whereas no band was amplified from the non-transgenic negative control and ddH_2_O blank control (Figure 4). Therefore, PCR analysis verified that 10 positive *cry2A* transgenic plants were successfully obtained.

### 2.4. RT-qPCR Detection of the cry2A Gene and the Copy Number Estimation in Transgenic Lines

Ten PCR-positive transgenic sugarcane lines were tested by RT-qPCR technique and the copy number of the *cry2A* gene was estimated. The RT-qPCR quantitative standard curve of *cry2A* gene was constructed using the following equation: *y* = −3.593*x* + 43.082, *R*^2^ = 0.994, where the y-axis represents the *C*_t_ value, the x-axis represents the logarithm of the initial template copy number. A good correlation between *C*_t_ values (18–40) and initial template copy number (10^1^–10^8^) was observed. According to the linear equation, x, the total copy number of the *cry2A* gene in the sample, was determined, and the number of exogenous *cry2A* copies of a single cell (Table 1) was calculated using the followed formula: Copies/genome = 10*^x^*/[25 ng × 10^−9^ × 6.02 × 10^23^/(10,000 × 10^6^ × 660)]. Table 1 shows that the *cry2A* gene copy number of different transgenic sugarcane lines varied, wherein three lines had two copies, five lines had three copies, and two lines had four copies.

### 2.5. cry2A Protein Expression in the Transgenic Lines

**Figure 5:**
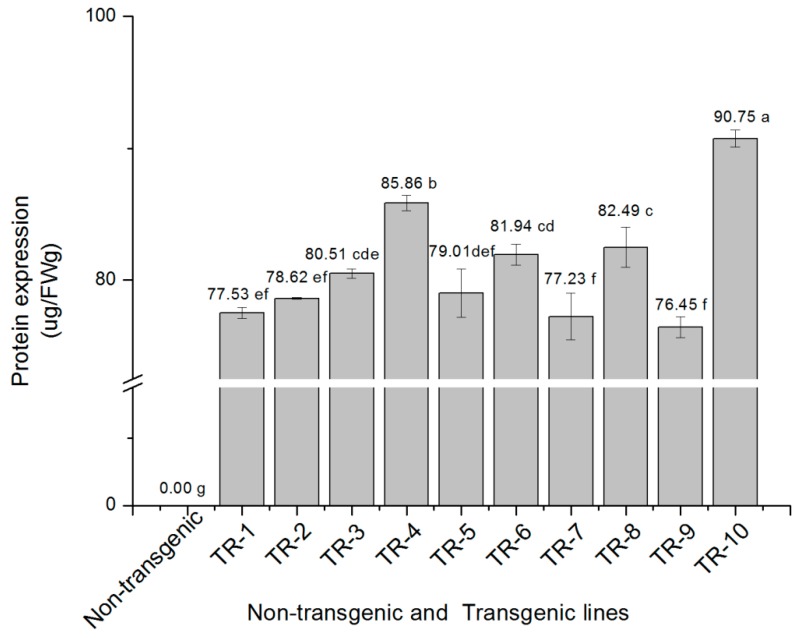
The *cry2A* protein expression in the leaves of non- transgenic and 10 different transgenic sugarcane lines detected by quantitative ELISA. The value is the average of three replicate experiments ± standard deviation (*n* = 3), and the different letters indicate significant difference at 0.05 level.

cry2A protein expression in the mature leaves of 10 transgenic sugarcane lines was quantitated by ELISA, and a standard curve was constructed using the Bt protein standard in the kit as the following equation: *y* = −1.156*x* + 3.865, *R*^2^ = 0.999, where the y-axis represents the OD_450_ absorbance, the x-axis represents the concentration of the Bt protein standard. The absorbance value correlated well with the protein standard concentration (R^2^ = 0.999). The amount of protein expression in the 10 samples was calculated according to the linear equation (Figure 5), which showed that the *cry2A* protein expression was observed in all 10 transgenic lines at levels within the range of 76.45–90.75 μg/FWg, of which three lines, namely, TR-4, TR-8, and TR-10 had higher protein expression levels (85.86, 82.49 and 90.75 μg/FWg, respectively) and the difference among the three lines was statistically significant.

### 2.6. Survey of Industrial and Agronomic Traits of the cry2A Transgenic Sugarcane Lines

According to protein expression and field performance, three *cry2A* transgenic sugarcane lines, TR-4, TR-8, and TR-10, were selected for the further field experiment using non-transgenic recipient ROC22 as a control, and plant height, stem diameter, brix, effective stalk number, and other indicators of industrial and agronomic traits at maturity were determined. The results were then subjected to univariate statistical analysis, and the results are shown in Table 2.

In the plant cane, when refer to the plant height of three transgenic lines, both TR-4 and TR-10 were slightly lower than the control, while the significant lower was observed in line TR-8. Although the stem diameters of the three lines were lower than the control, but not statistically significant. The brix (of the three lines) was higher than that of the control, although not statistically significant. The lines TR-4 and TR-8 had slightly lower number of effective stalk per hectare than the control, while line TR-10 is slightly higher than the control, but both had no significant difference. The theoretical sugar yield of TR-4 and TR-10 was comparable to that of the control, whereas TR-8 was significantly lower than the control.

In the ratoon cane, the height and stem diameter of the three transgenic lines were lower than the control, however, only the plant height of TR-8 and the stem diameter of TR-10 were significantly lower than the control. The brix of the three transgenic lines was higher than that of the control, and TR-8 and TR-10 were significantly higher than the control. Although the number of effective stalk per hectare of TR-8 and TR-10 were higher than that of the control, this difference was not statistically significant. Similar to that in the plant cane, the theoretical sugar yield of TR-4 and TR-10 were comparable to that of the control, while significantly lower was observed in TR-8. Compared to the plant cane, the brix of the transgenic lines in the ratoon cane increased, of which TR-8 and TR-10 increased by more than 1.0, while the control ROC22 decreased by 0.43.

### 2.7. Insect Resistance Identification of the cry2A Transgenic Sugarcane Lines

**Figure 6:**
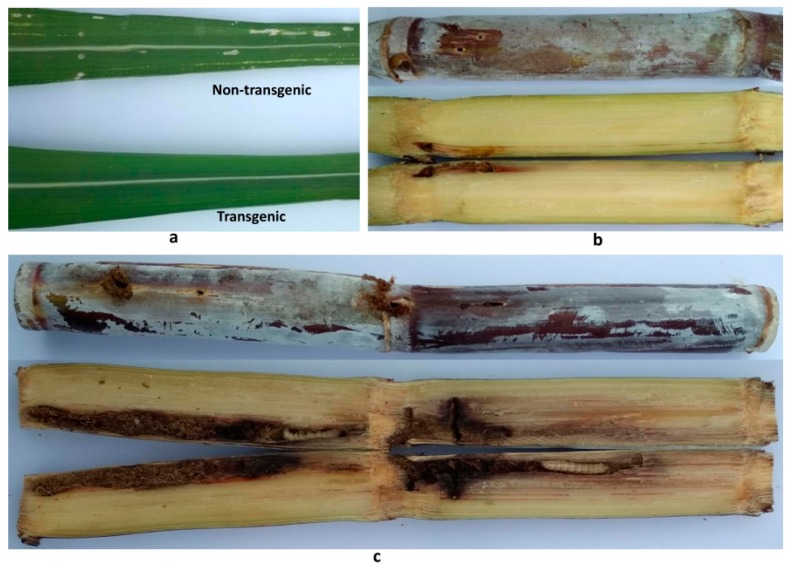
Stem borer damage in sugarcane under natural field conditions: (**a**) symptoms of the transgenic and non-transgenic sugarcane leaf; (**b**) symptoms of the transgenic sugarcane stem (only in cortex); and (**c**) symptoms of non-transgenic sugarcane stem.

Under natural field conditions, the percentage of borer-infested plants of the three lines and the control ROC22 in the plant cane and ratoon cane were investigated. The leaves and stems of the transgenic sugarcane lines showed more pronounced insect resistance compared to that in the non-transgenic control ROC22 (Figure 6). The survey results (Table 3) showed that the percentage of borer-infested plants in the three transgenic lines was lower than the control. In the plant cane, the percentage of borer-infested plants in the TR-10 line was only 26.67%, but as high as 80.0% in the control, and the difference was statistically significant. Although the TR-4 and TR-8 lines compared to the control did not reach significant level, these were only 36.67% and 53.33%, respectively. After one year of ratooning, the percentage of borer-infested plants decreased in the three transgenic lines, but slightly increased in the control, and the percentage of borer-infested plants was 30.0% and 16.67% in TR-4 and TR-10 lines, respectively, which was significantly lower than the 83.33% of the control. Especially, the transgenic lines affected by stem borers only incurred damages in the cane stem cortex (Figure 6b), whereas the control line exhibited more serious damage with injuries in the entire stem (Figure 6c).

## 3. Discussion and Conclusions

Sugarcane is a perennial crop and, to save costs, ratooning is usually conducted for over three years or even up to five years or more, during which sucrose content reduction and wind broken stalk increases can be caused by stem borer [4,5]. Arencibia et al. (1997) transformed the *cry1A(b)* gene into sugarcane by the cell electroporation, and improved the stem borer resistance [8]. Arvinth et al. (2010) introduced the *cry1Ab* gene into sugarcane, which significantly reduced the percentage of dead heart sugarcane seedlings [22]. The *GNA* gene was integrated into sugarcane genome via *Agrobacterium*-mediated transformation by Zhangsun et al. (2007), and the results showed that transgenic sugarcane plants had a significant resistance to the woolly aphid [25]. Falco et al. (1997) introduced the soybean bowman-birk inhibitor into sugarcane callus using particle bombardment, and it demonstrated that, compared to larvae fed on leaf tissue from untransformed ones, the growth of larvae feeding on leaf tissue from transgenic plants was significantly retarded, however the retardation was not sufficient to prevent the “dead heart” symptom [30]. Weng et al. (2011) [28] and Gao et al. (2016) [29] introduced the *cry1Ac* gene into different sugarcane varieties, and the transgenic sugarcane plants showed much better resistance to stem borer than the non-transgenic ones. ROC22, the most widely cultivated cultivar accounting for more than 60% of Chinese sugarcane acreage in the past 15 years, was used as the receptor in this study. A field test comparing the three transgenic lines with non-transgenic control found that the percentage of borer-infested plants of the transgenic lines in the ratoon cane decreased compared to that in the plant cane, whereas contrarily, slightly increased in the control. In addition, compared to the control, line TR-4, exhibiting markedly lower in frequency of borer-infested plants in the ratoon cane (30.0% vs. 83.3%) and much lower in plant cane (36.67% vs. 80.0%) indicating that the stress of stem borers gradually shifted to non-transgenic control.

Assessment of copy number of transgenic lines is essential to phenotypic studies and investigations on genetic stability. The traditional method for copy number identification is the Southern blot, which is highly cumbersome and strongly operation dependent [44], and various external factors may influence visualization of hybridization bands and thus are often underestimated. Previous research has shown that Southern blotting was not able to accurately determine the number copy numbers of exogenous genes in sugarcane b, whereas RT-qPCR is characterized by high specificity and high sensitivity, and thus more accurate [29,45]. RT-qPCR has been widely used to identify exogenous gene copy number [44,45,46,47,48,49], even for transgene copy number from 3 to >50 [45]. Sugarcane has a complex genetic background and is a highly heterogeneous polyploid or aneuploid crop, with genome sizes of up to 10 Gb [50]. In the present study, an RT-qPCR assay standard curve for the *cry2A* gene with a slope of −3.593 and a correlation coefficient of 0.994 was established, which indicated that PCR amplification efficiency and *C*_t_ values correlate well with the initial template copy number. Based on the standard curve, the *cry2A* gene copy number in the 10 transgenic sugarcane lines was determined, which revealed that the copy number of each transgenic line did not exceed four copies, which is discrepant to the findings of our previous study on *cry1Ac* transgenic sugarcane [29]. These may be related to different exogenous genes introduced and different genotypes of receptor materials.

In the present study, ELISA was used to quantitatively determine the cry2A protein expression levels in the leaves of 10 obtained transgenic lines, which ranged from 76.45 to 90.75 μg/FWg, with significant differences in some lines. However, no clear linear relationship between protein expression and copy number was observed, which was similar to that observed in previous studies [51,52,53]. The expression of exogenous Bt protein can effectively improve the insect resistance of transgenic plants [18,54]. Weng et al. (2011) introduced a modified *cry1Ac* gene into sugarcane cultivars ROC16 and YT79-177 by particle bombardment, and 17 transgenic plants were positive for Western blot. It also demonstrated that the expression of water-soluble proteins in leaves ranged from 2.2 ng/mg to 50 ng/mg, and when the expression exceeded 9 ng/mg (9 μg/FWg), insect resistance was observed, with the content of cry1Ac protein in transgenic sugarcane positively correlated with its insect resistance [28]. Arvinth et al. (2010) found that the total soluble cry1Ab protein expression in the obtained transgenic sugarcane leaves ranged from 0.007% to 1.73%, and protein expression was negatively correlated to the percentage of dead heart seedlings [22]. In our previous research, the cry1Ac protein expression in *cry1Ac* transgenic sugarcane leaves ranged from 0.85 to 70.9 μg/FWg, and the higher the protein expression, the lower the percentage of borer-infested plants, which exhibited a significant negative correlation [29]. Here, again, we observed that the higher the protein expression, the better the insect-resistant effect, which is consistent with the results of our previous investigation [29] and with other reports [22,28].

Weng et al. (2011) generated a ubiquitin (ubi) initiated *cry1Ac* transgenic sugarcane, and the assessment of industrial and agronomic traits showed that the agronomic traits such as plant height and stem diameter were greatly affected. However, the industrial indicators such as sucrose content and brix exhibited no significant difference compared to the control [28]. Our group previously conducted a field survey on double 35 s initiated *cry1Ac* transgenic sugarcane cultivar FN15, and it showed that only 2 of 14 transgenic lines had slightly greater plant heights than the control, although not statistically significant, whereas the other lines were lower than the control, and all the stem diameters (of the transgenic lines) were lower than that of the control. However, both higher and lower brix than that of the control was observed, and the calculated theoretical sugar yields (of the transgenic lines) were all lower than that of the control though three lines are unobvious [29]. Wang et al. (2017) introduced *cry1Ab* gene into sugarcane cultivar ROC22 by *Agrobacterium*, and investigated the industrial and agronomic traits of five single-copy transgenic lines. The result showed that plant height, stem diameter, brix, effective stalk number of several transgenic lines was only slightly lower than that of the control, while calculated theoretical sugar yield was significantly lower than that of the control [23]. Besides, a three-year field performance trial of transgenic sugarcane with *npt II* gene showed a reduction in growth and cane yield, but, when individual events were analyzed separately, the yields of several transgenic events were comparable to that of no transformants [53]. The present study conducted on plant cane and ratoon cane in the field using the obtained transgenic lines TR-4, TR-8, and TR-10, and their theoretical sugar yields were all lower (9.33–11.07 t/ha for plant cane, and 9.70–10.87 t/ha for ratoon cane) than that of the control (11.26 and 10.96 t/ha). It indicates that the introduction of exogenous *cry2A* gene into sugarcane increased stem borer resistance and reduced the percentage of infested plants, while the expression of the Bt protein consumes energy, thereby resulting in a decrease in sugar yield in generally, though comparable sugar yield of transgenic lines can be obtained, such as TR-4 and TR-10 in this study, which is in line with the results of two previous researches [29,53].

In conclusion, the introduction of the *cry2A* gene via particle bombardment produces the transgenic lines with obviously increased stem borer resistance and comparable sugar yield, providing a practical value in direct commercial cultivation, and crossbreeding for ROC22 has been used as the most popular elite parent in various breeding programs in China.

## 4. Materials and Methods

### 4.1. Materials

The plant expression vector pGreenII0229 was obtained from John Innes Center in Norwich, Norfolk, UK, and the clone 2AST1305.1 containing the *cry2A* gene was a gift from Professor Illimar Altosaar of the University of Ottawa in Canada. The pGreen plasmid can help plant genetic transformation because it was a versatile and flexible binary vector [55], and the pGreenII0229 vector contains the *bar* gene as the screening marker gene. The receptor material used for genetic transformation was ROC22, the most widely cultivated sugarcane cultivar in mainland China, which was provided by Key Laboratory of Sugarcane Biology and Genetics and Breeding, Ministry of Agriculture, China.

### 4.2. Plant Vector Construction of the cry2A Gene

The *cry2A* gene plant expression vector was constructed using the directional cloning strategy. First, plasmid DNA of the 2AST1305.1 cloning vector that harbored the exogenous *cry2A* gene was digested with restriction endonucleases *Eco*R I and *Hin*d III. The exogenous gene expression cassette containing the ST-LS1 promoter, the *cry2A* gene, and the *nos* terminator was recovered. Meanwhile, the plasmid DNA of the plant expression vector pGreenII0229 was digested with restriction enzymes *Eco*R I and *Hin*d III, and the target fragment containing the *bar* gene as a screening marker gene was recovered. Finally, the two recovered fragments were ligated with T4-DNA ligase to obtain a new *cry2A* gene plant expression vector pGcry2A0229.

### 4.3. Transformation and Screening

Shoots of ROC22 sugarcane plants that showed robust growth in the field were selected. The leaves were collected from the shoots and disinfected with 75% alcohol, and the outer leaf sheaths were stripped under aseptic conditions. Then, the heart lobe above the growth point was removed and sliced into about 2-mm thick discs, cultured in the dark at 26–28 °C for 2–4 weeks, and then subjected to particle bombardment transformation after callus generation [29]. Before bombardment, the tungsten particles (Bio-Rad, Foster City, CA, USA, 0.7) were coated by the plasmid of pGcry2A0229 DNA as the micro-bombs, with 1.0 μg of DNA each bombardment. The operation was performed according to the protocol of the PDS-1000/He gene gun (Bio-Rad, Hercules, CA, USA). The bombarded and transformed material was restored culture in subculture medium, then subjected to a screening culture using 0.8 mg/L PPT according to our preliminary experiment, until the plantlets had differentiated, which refers to literature for details [29]. Once developing roots, the plants were transplanted into a nutrient pot. Upon reaching a height of about 10 cm and on a sunny day, the plants were sprayed with 3.0‰ Basta solution (*v*/*v*) [29]. Calli were inducted on medium consisted of MS, 3.0 mg/L 2,4-D, 30 g/L sucrose, and 6.0 g/L agar powder, at a pH of 5.8. The subculture medium comprised MS, 2 mg/L 2,4-D, 30 g/L sucrose, and 6 g/L agar powder, at a pH of 5.8. The differentiation medium included MS, 1.5 mg/L 6-BA, 1.0 mg/L KT, 0.2 mg/L NAA, 30 g/L sucrose, and 6 g/L agarose, at a pH of 5.8. The rooting medium consisted of ½ MS, 0.2 mg/L 6-BA, 3 mg/L NAA, 60 g/L sucrose, and 5.5 g/L agarose, at a pH of 5.8.

### 4.4. DNA Extraction and Primer Design

Genomic DNA was extracted from young leaves of resistant plants that survived the PPT and Basta screening and non-transgenic ROC22 negative control plants using a modified CTAB method [56]. Based on the *cry2A* gene sequence, Primer Premier 5 software was used to design PCR and RT-qPCR primers. The PCR primers were as follows: 2ast1178s: 5′-AACAGGCAACAACCCATAGAGG-3′ and 2ast1798r: 5′-AGGGAGCCCACCTTCTTGAG-3′, and the resulting amplified fragment was 620 bp in size. The RT-qPCR primers were as follows: forward primer: 5′-CAACCAGCAGGTGGACAACTT-3′, reverse primer: 5′-AAGAGCTGCTGCATGGTGTTC-3′, and probe: 5′-CTCAACCCGACCCAGAACCCGG-3′.

### 4.5. PCR Amplification of Putative Transgenic Sugarcane Lines

Using non-transformed ROC22 as the negative control, pGcry2A0229 plasmid DNA containing the *cry2A* gene as the positive control, and ddH_2_O as a blank control, amplification and identification were performed using an Eppendorf 5331 PCR instrument (Eppendorf, Hamburg, Germany). Each PCR amplification system consisted of the following reagents: 2.5 μL of 10 × PCR buffer (Mg^2+^ Plus), 2.0 μL of a dNTP mixture (2.5 mmol/L each), 1.0 μL of the DNA template (50.0 ng/μL), 1 μL each of the upstream and downstream primers, 0.25 μL of Taq DNA polymerase (5 U/μL), and topped up to 25.0 μL with ddH_2_O. The reaction conditions were as follows: pre-denaturation at 95 °C for 5 min; followed by 30 cycles of denaturation at 95 °C for 30 s, annealing at 57 °C for 30 s, and extension at 72 °C for 40 s; and a final extension at 72 °C for 10 min. After amplification, the PCR products were electrophoresed on a 1.5% agarose gel and photographed using a gel imaging system.

### 4.6. Copy Number Calculation in Transgenic Sugarcane Lines by RT-qPCR

The *cry2A* gene was quantitatively detected in the PCR-positive transgenic sugarcane lines using the designed and synthesized RT-qPCR primers. The fluorescence quantitative PCR instrument was an ABI PRISM 7500 Sequence Detection System (Foster City, CA, USA). The total volume of the detection system was 25.0 μL, which contained 12.5 μL of a FastStart Universal Probe Master Mix, 1.0 μL of gDNA (25.0 ng/μL), 1.0 μL (10.0 μmol/L) of the forward primer, 1.0 μL (10.0 μmol/L) of the reverse primer, 0.2 μL (10.0 μmol/L) of probe, and then topped up to a final volume of 25.0 μL with ddH_2_O. The amplification conditions were as follows: 50 °C for 2 min; 95 °C for 10 min; 40 cycles of 95 °C for 15 s and 60 °C for 1 min; and a final cycle of 95 °C for 15 s, 60 °C for 15 s, and 95 °C for 15 s. Three replicates were used for each sample. At the same time, gradient dilutions of 10^8^, 10^7^, 10^6^, 10^5^, 10^4^, 10^3^, 10^2^, and 10^1^ copies/μL were prepared using pGcry2A0229 plasmid DNA. Plasmid copy number was calculated using the following equation: Plasmid copy number (copies/μL) = 6.02 × 10^23^ copies/mol × plasmid concentration (g/μL)/plasmid molecular weight (g/mol)/660 [57]. After the reaction, using log(plasmid copy number) as x-axis and the *C*_t_ value as y-axis, a standard curve was generated using the formula *y* = k*x* + b. Further, based on the *C*_t_ value (y) and linear equation, the total copy number (10*^x^*) of the *cry2A* transgenic lines was determined, and then the single cell copy number of each sample was calculated using the following formula: Copies/genome = 10*^x^*/[25 ng × 10^−9^ × 6.02 × 10^23^/(10,000 × 10^6^ × 660)] [58].

### 4.7. Quantitative ELISA of the cry2A Protein in Transgenic Sugarcane Lines

The *cry2A* protein in the leaves of PCR-positive transgenic sugarcane plants was detected using double-antibody sandwich enzyme linked immunosorbent assay (ELISA). Non-transformed ROC22 plants were used as the negative control and ddH_2_O as a blank control. Gradient dilutions of cry2A protein reference standards in a Qualiplat kit for *cry2A* purchased from Envirologix (Portland, OR, USA) were prepared, with the y-axis representing the OD_450_ absorbance and the x-axis representing the Bt standard protein concentration to construct a standard curve. Quantitative ELISA was conducted according to the protocol provided in the *cry2A* protein assay kit. Three replicates of each sample were prepared.

### 4.8. Field Trial Design and Assessment of Phenotype Traits of the Transgenic Sugarcane Lines

Three *cry2A* transgenic sugarcane lines with good performance in the field were selected used for further investigation, and non-transgenic ROC22 was used as the control in the field experiment. The experiment followed a randomized block design that consisted of triplicates. The length of the plot was 8.0 m, three rows with a row spacing of 1.3 m were used, the plot area was 31.2 m^2^, and 13 buds per meter length. The present study applied common fertilizers at amounts routinely used in the sugarcane field: 345.0 kg/ha of nitrogen fertilizer (N), 240.0 kg/ha of phosphate fertilizer (P_2_O_5_), and 360.0 kg/ha of potassium fertilizer (K_2_O), coupled with normal field management. The industrial and agronomic traits including plant height, stem diameter, brix, effective stalk number, and percentage of borer-infested plants at maturity were investigated, and 20 plants in each plot as the biological repeats in the plot were measured. At the same time, 5 m long and more evenly distributed sections in each plot were selected, and the effective stalk number was counted. The theoretical cane yield per mu and sugar yield per mu were calculated according to the formulae [59]:

Theoretical cane yield = Plant height × Stem diameter^2^ × 0.785/1000 × Effective stalk number
Sucrose content (%) = Brix × 1.0825 − 7.703
Theoretical sugar yield = Theoretical cane yield × Sucrose content (%)
DPS analysis software and Tukey method were used for statistical analysis of the collected data.

## Figures and Tables

**Table 1:** Estimated *cry2A* gene copy number of different transgenic lines.

Line	*C*_t_ I	*C*_t_ II	*C*_t_ III	*C*_t_ Mean	Copy Number
TR-1	29.96	29.84	30.19	30.00 ± 0.10	1.92
TR-2	29.10	29.39	29.22	29.24 ± 0.08	3.13
TR-3	29.78	30.06	30.20	30.01 ± 0.12	1.90
TR-4	29.95	30.11	30.09	30.05 ± 0.05	1.86
TR-5	29.08	28.97	29.10	29.05 ± 0.04	3.53
TR-6	29.75	29.67	29.63	29.68 ± 0.04	2.35
TR-7	29.70	29.39	29.70	29.60 ± 0.10	2.49
TR-8	29.48	29.51	29.62	29.54 ± 0.04	2.58
TR-9	29.68	29.68	29.78	29.72 ± 0.03	2.30
TR-10	30.00	29.10	29.48	29.53 ± 0.26	2.60
Non-transgenic	37.97	38.22	40.03	38.74 ± 0.65	0.01

**Table 2:** Industrial and agronomic traits of different *cry2A* transgenic sugarcane lines during the plant and ratoon cane.

Crop Season	Line	Plant Height, H	Stem Diameter, D	Brix, Bx	Effective Stems (stem/ha)	Theoretical Sugar Yield (t/ha)
Plant cane	TR-4	270.23 ± 3.23 ^a^	2.33 ± 0.16 ^a^	20.74 ± 0.26 ^a^	64,989.74 ± 1982.53 ^a^	11.02 ± 0.34 ^a^
	TR-8	240.27±1.88 ^b^	2.30 ± 0.07 ^a^	20.39 ± 0.36 ^a^	64,989.74 ± 799.89 ^a^	9.33 ± 0.13 ^b^
	TR-10	258.67 ± 5.06 ^a^	2.34 ± 0.12 ^a^	20.33 ± 0.32 ^a^	69,692.95 ± 709.90 ^a^	11.07 ± 0.18 ^a^
	Non-transgenic	272.70 ± 4.71 ^a^	2.38 ± 0.06 ^a^	19.98 ± 0.30 ^a^	66,700.00 ± 1385.47 ^a^	11.26 ± 0.19 ^a^
Ratoon cane	TR-4	271.13 ± 8.05 ^a,b^	2.50 ± 0.04 ^a,b^	20.91 ± 0.47 ^a,b^	54,646.15 ± 1397.39 ^a^	10.87 ± 0.28 ^a^
	TR-8	248.57 ± 3.48 ^b^	2.46 ± 0.02 ^a,b^	21.69 ± 0.11 ^a^	52,084.61 ± 1597.40 ^a^	9.70 ± 0.21 ^b^
	TR-10	264.33 ± 5.02 ^a,b^	2.36 ± 0.07 ^b^	21.85 ± 0.15 ^a^	58,061.53 ± 1597.39 ^a^	10.73 ± 0.17 ^a^
	Non-transgenic	279.43 ± 2.96 ^a^	2.66 ± 0.10 ^a^	19.85 ± 0.53 ^b^	51,230.77 ± 2091.48 ^a^	10.96 ± 0.20 ^a^

Data followed with the different letters (^a^ and ^b^) indicate significant difference at 0.05 level, but the same letter (^a^ or ^b^) indicates that the difference is not significant.

**Table 3:** The percentage of borer-infested plants of different *cry2A* transgenic sugarcane lines in plant and ratoon cane.

Line	Percentage of Borer-Infested Plants (%)	
	Plant Cane	Ratoon Cane
TR-4	36.67 ± 12.02 ^a,b^	30.00 ± 11.55 ^b^
TR-8	53.33 ± 17.64 ^a,b^	50.00 ± 10.00 ^a,b^
TR-10	26.67 ± 5.77 ^b^	16.67 ± 3.33 ^b^
Non-transgenic	80.00 ± 6.67 ^a^	83.33 ± 6.67 ^a^

Data followed with the different letters (^a^ and ^b^) indicate significant difference at 0.05 level, but the same letter (^a^ or ^b^) indicates that the difference is not significant.

## Abbreviations

ELISA	Enzyme-linked immuno sorbent assay
CTAB	Cetyltrimethyl ammonium bromide
G418	Geneticin
Hyg	Hygromycin
KT	Kinetin
MS	Murashige and shoog medium
NAA	Naphthalene acetic acid
PPT	Phosphinothricin
2,4-D	2,4-Dichlorophenoxy acetic acid
6-BA	6-Benzyladenine
